# Pharyngeal Gonorrhoea: The Willingness of Australian Men Who Have Sex with Men to Change Current Sexual Practices to Reduce Their Risk of Transmission—A Qualitative Study

**DOI:** 10.1371/journal.pone.0164033

**Published:** 2016-12-19

**Authors:** Sandra Walker, Clare Bellhouse, Christopher K. Fairley, Jade E. Bilardi, Eric P. F. Chow

**Affiliations:** 1 Central Clinical School, Faculty of Medicine, Nursing and Health Sciences, Monash University, Melbourne, VIC, Australia; 2 Melbourne Sexual Health Centre, Alfred Health, Melbourne, VIC, Australia; 3 Department of General Practice, The University of Melbourne, Melbourne, VIC, Australia; Universidade Estadual de Maringa, BRAZIL

## Abstract

**Background:**

The pharynx is a common site of gonorrhoea among men who have sex with men (MSM) and may serve as a reservoir for infection, with saliva implicated in transmission possibly through oral sex, kissing, and rimming. Reducing sexual activities involving saliva may reduce pharyngeal gonorrhoea. This study aimed to explore MSM’s views and knowledge of pharyngeal gonorrhoea and their willingness to change saliva transmitting sexual practices. MSM were also asked their views on using alcohol-containing mouthwash to potentially reduce transmission.

**Methods:**

Using a qualitative descriptive approach, 30 MSM who were part of a larger study (GONE) conducted at the Melbourne Sexual Health Centre agreed to take part in semi-structured interviews between 14^th^ May and 8^th^ September 2015. The 10 interviews conducted face to face and 20 by telephone, lasted between 20–45 minutes. Data were analysed using qualitative content analysis.

**Results:**

Most men considered pharyngeal gonorrhoea to be a non-serious sexually transmitted infection and attributed transmission primarily to oral sex. Almost all men reported they would not stop kissing, oral sex, or consider using condoms for oral sex to reduce their risk of pharyngeal gonorrhoea. Kissing and oral sex were commonly practised and considered enjoyable low risk sexual activities. Men were more likely to consider stopping sexual activities they did not enjoy or practice often, in particular insertive rimming. If proven effective, the majority of men reported they would use alcohol-containing mouthwash to reduce or prevent their risk of pharyngeal gonorrhoea.

**Conclusion:**

Findings from this study suggest MSM are unlikely to stop saliva transmitting sexual practices they enjoy and consider low risk. Men would, however, consider using alcohol-containing mouthwash if found to be effective, highlighting the importance of exploring innovative strategies to reduce pharyngeal gonorrhoea.

## Introduction

Gonorrhoea is a very common bacterial sexually transmitted infection (STI) among men who have sex with men (MSM) in Australia and other developed countries[1–8] In Australia, the rate of notified gonorrhoea in males has nearly doubled over the past 10 years from 53.1 per 100,000 in 2005 to 98.6 per 100,000 in 2014 [8] with the majority of cases MSM.

The primary sites for gonorrhoea in MSM include the pharynx, urethra, and ano-rectum [9–11]. Gonorrhoea can occur at all three sites or at a single site, with the pharynx being the most common [3, 10]. The prevalence of pharyngeal gonorrhoea in MSM ranges from 3% to 15% worldwide. [12–14].

Potential antibiotic resistance has raised concerns that we may see further rises in gonorrhoea among MSM [12–16] with possible associated rises in HIV acquisition [17–22].

Among MSM, the pharynx is a common site for gonococcal infection with past epidemiological studies showing associations with oral sex [1, 4, 10, 23, 24], kissing and rimming (oro-anal sex) [5]. In a cohort study of 1427 HIV-negative MSM in Sydney, Australia, Templeton *et al*, (2010), reported young age, high numbers of sexual partners, oro-anal sex and recent sexual contact with gonorrhoea were associated with incident pharyngeal gonorrhoea [5]. In a study by Rosenberger *et al*. (2011) of 24,787 MSM in the United States, the most common practices men reported in their recent sexual encounter with another man were kissing on the mouth (75%), oral sex (73%) and mutual masturbation (68%) [25]. Furthermore, the majority of MSM consider kissing (75%), insertive (64%) and receptive oral sex (70%) to be very exciting sexual activities [26]. While the significance of 'deep kissing' has to date not been assessed, gonococci was found in the saliva in the anterior part of the oral cavity, and kissing was implicated as a possible route of transmission [27]. The transmission of gonorrhoea by kissing has also been considered by Wilmot *et al*.[28]. However using a condom for oral sex to prevent STIs has been found to be unfavourable to men due to the taste and sensation of condoms [29].

In a recent Australian study of MSM with pharyngeal gonorrhoea, gonorrhoea was detected in the saliva of 43% (6 of 14) of men who tested positive from a pharyngeal swab, by culture.

[7]. Saliva use as a lubricant for anal sex and fingering is common among MSM and it has been reported that both practices are risk factors for gonorrhoea [30, 31]. Chow *et al*, (2016) in their crossectional study of 1312 Australian MSM in Melbourne, Victoria, found 68.5% of MSM reported using saliva as a lubricant for anal sex [31].

The pharynx has been reported to be a reservoir for *N*.*gonorrhoeae* which may act as a route for transmission of gonorrhoea to other sites [23] and the key site to prevent further spread of antibiotic resistance [32]. Given that there is some evidence to suggest *N*.*gonorrhoeae* may be transmitted through saliva, and that saliva is commonly used in sexual practices enjoyed by MSM such as oral sex, exploring novel approaches to reduce transmission from the pharynx is worthy of consideration.

Listerine was touted by its original manufactures to be a cure for gonorrhoea [33], and in a recent study the authors report alcohol-containing mouthwash is effective in inhibiting gonorrhoea in the pharynx [34]. To our knowledge there has been no previous research investigating the effectiveness or acceptability of a mouthwash to reduce pharyngeal gonorrhoea detection. It is unknown what MSM know about the sexual practices that lead to pharyngeal gonorrhoea, and whether they consider saliva use might be implicated in transmission.

The aim of this study was to explore MSM’s views and knowledge of gonorrhoea and their willingness to change sexual practices to reduce transmission. We also explored men’s views on using alcohol-containing mouthwash to potentially reduce the risk of transmission.

## Method

This study has been reported in accordance with the Consolidated criteria for Reporting Qualitative research (COREQ) guidelines [35].

### Ethics Statement

Ethics approval for this study was granted by the Alfred Hospital Ethics Committee, Victoria, Australia (number 544/14) on the 30^th^ January 2015.

### Study Design

A Qualitative Descriptive (QD) approach was used in this research study. QD is a pragmatic rather than theory-driven approach which is based on expert knowledge, linkages to the works of others in the field and the clinical experience of the research group [36]. QD is commonly used in healthcare research as it allows researchers to address questions of specific clinical relevance and aims to provide a description of events or participant experiences by staying close to the data and the informant’s point of view, rather than providing an interpretative or theory based analysis [36]. A QD approach is also useful in providing preliminary insight into a largely unexplored issue or topic, informing the development of future interventions or needs assessments, in particular for vulnerable population groups or when resource constraints must be considered [36, 37].

### Recruitment

Participants were recruited as part of a larger gonorrhoea study among MSM, the **GON**orrhoea **E**radication study (GONE). The GONE study was conducted at the Melbourne Sexual Health Centre (MSHC), the largest urban sexual health centre in Victoria, Australia, which has a high caseload of MSM. Pharyngeal swabs were collected from MSM and tested by nucleic acid amplification test (NAAT) for gonorrhoea. The study aimed to examine the risk factors, such as kissing, associated with pharyngeal gonorrhoea in MSM. In the GONE study men completed a 29-item multiple choice questionnaire between 30^th^ March and 23^rd^ September 2015.

As part of the questionnaire men were asked whether they would like to participate in an interview to explore their views on pharyngeal gonorrhoea and strategies that might work to reduce the risk of transmission. Participants could either select ‘yes/maybe’ or ‘no’ to this question. Those who selected ‘yes/maybe’, were contacted within a week of completing the questionnaire by a research assistant to determine eligibility for the study and if eligible, to email them a participant information form. Eligibility included: Men who have sex with men, aged 16 years and over, with a good understanding of written and verbal English who were willing to be contacted by telephone and email.

Men who were eligible and confirmed their interest in the study were scheduled for an interview, where possible, within a week after interest was confirmed to optimize study retention. A reminder text message was sent the day before the interview. The interview schedule was devised by EPFC, JB and SW and pilot tested on four staff members working in the area of gonorrhoea research, prior to use. The interview explored knowledge of pharyngeal gonorrhoea, sexual behaviours men prefer to engage in with partners and why, saliva use in sexual practice, changing sexual practices to reduce the risk of pharyngeal gonorrhoea transmission, and views and acceptability of using alcohol-containing mouthwash to potentially reduce pharyngeal gonorrhoea.

Table 1 outlines the sampling framework.

**Table 1:** Sampling framework.

Sampling framework
Men who have sex with men
Single and men who are in a relationship
Men with low and high numbers of sexual partners in the last 3 months
Recruited from Melbourne Sexual Health Centre, Australia
Men with a recent positive and negative diagnosis of pharyngeal gonorrhoea
Broad range of ages
***Revised during data collection to include*:**
Increased number of men with a positive diagnosis of pharyngeal gonorrhoea in the last 3 months (to increase the numbers of positive testing men so as to increase the sample distribution for diagnosis and to compare experiences of men testing positive and negative)

### Data collection

Men had the option of being interviewed face to face at MSHC or by telephone. All interviews were conducted between 14^th^ May and 8^th^ September 2015 and involved a once off single interview. In the initial interviewing phase, two researchers (SW and JB) were present for the first three interviews with JB note taking and SW interviewing. SW is an experienced sexual health researcher and health psychologist (Doctorate in Health Psychology) with previous experience interviewing MSM on sensitive topics and JB is an experienced qualitative sexual health researcher (PhD in Public Health) accustomed to interviewing participants about sexual health issues. SW and JB had no prior relationship with the participants. Both researchers had a good understanding of the epidemiology of pharyngeal gonorrhoea. Participants were informed that the research study was being undertaken in an effort to better understand men’s willingness to reduce their risk of pharyngeal gonorrhoea.

The presence of both researchers initially allowed for discussion and cross checking of the data emerging from interviews, refinement of the interview schedule and reduced subjective bias of emergent themes. Permission was obtained from the participant to have both researchers present at the time of interview and participants were aware that the research was being conducted on behalf of MSHC. Following initial refinement of the interview schedule, it was deemed no longer necessary to have both researchers present for all interviews. All remaining interviews were conducted by SW alone and JB consulted throughout the data collection process.

All interviews were conducted in a private room at MSHC. Participants who chose to have their interview face to face, completed and returned their consent form at the time of interview and were offered the option of a hard copy for their records. Men who undertook their interview by telephone were asked to provide verbal consent over the telephone as it was not practical to obtain written consent for this method of interview. Prior to commencement of interviewing by telephone, SW read aloud the consent form, signed it on behalf of the participant and offered to send him a hard copy by mail or email. The interview schedule was semi-structured, allowing for open ended questions and contained themes and prompts derived from the literature and expert clinical knowledge to guide discussion. Demographic information were collected through the larger GONE study questionnaire. Consent for the collection of this information was given through participation in the larger GONE study and men were aware their demographic data would be used for the purpose of this study.

All interviews were digitally recorded and transcribed verbatim. Following each interview a participant summary was written by SW and added to each transcript. Participants were offered the option of receiving their interview transcripts for member checking and editing. Four men elected to review their transcript and none requested amendments. Participants were offered a $50 supermarket gift voucher in appreciation of their time.

### Data pre-analysis

Throughout the data collection process regular meetings were held between SW and JB to discuss the interview data, emerging themes and the sampling framework. After completion of 10 interviews, the interview data and sampling framework was reviewed by SW and JB to determine whether further refinements or questioning was required. At this time it was decided to purposively sample more young men and men with a recent (in the last three months) diagnosis of pharyngeal gonorrhoea to ensure a broad range of men’s views was collected. Purposive sampling is a non-probability sampling technique which aims to purposively recruit people with certain characteristics of interest, and not pre-determined numbers of sub-groups and this method is commonly used in qualitative research [38].

SW and JB continued to meet regularly to review transcripts and themes as the interviewing process continued and after 30 interviews were complete it was decided saturation point had been met and no further interviews were required.

### Data Analysis

Qualitative content analysis was used in the analysis of the data. Content analysis is a method frequently used in qualitative descriptive studies to provide a descriptive summary of the data content using modifiable coding systems that correspond to the data collected. Analysis is undertaken by applying codes to information derived from the data and sorting the data into groups or conceptual categories [36, 39].

All interview transcripts were initially read several times by SW who began by firstly noting down preliminary codes. Using a coding tree, emerging concepts were each allocated a code which were then categorised into broader themes and subthemes. Following the initial coding, JB reviewed a subset of transcripts independently, using the same process of coding and theme identification. Both researchers met to compare and discuss their coding and theme categorisations with no major differences evident between researchers. A third researcher (CB) also found consensus in emerging themes.

All transcribed interviews were then imported into NVivo 10 [40] for data management. Using the initial coding framework as a guide, each transcript was again re-read by SW and the coding framework and participant responses under each theme and subtheme compared for similarities and differences. The final analysis was examined and confirmed by JB in a comprehensive review of the data. Frequency analyses of demographic, sexual behaviour and diagnosis were conducted using STATA 13 (Stata Corp, College Station, TX, USA).

## Results

A total of 243 men from the GONE study indicated they would be willing to be contacted for an interview. Of those men the first 133 were called by a research assistant, of which 66 did not answer the phone call, 12 were no longer interested in the interview, 13 were interested in the interview but did not confirm an interview time, and the remaining 42 were interested and scheduled a time for an interview. Men were contacted a maximum of 3 times with no further contact made if they were unable to be reached. Of the 42 men scheduled for an interview, 12 did not present and 30 attended for interview. Of the 30 interviews, 10 were conducted face to face and 20 by telephone. No further contact was made with the men who ticked ‘yes/maybe’ to an interview on the GONE questionnaire as saturation point had been met after 30 interviews.

Interviews ranged in length from 20 to 45 minutes. The median age of men was 32 [range 20–73]. The majority of men (n = 22, 73%) had been diagnosed with gonorrhoea (rectum, urethra or pharynx) in their lifetime and a third had a diagnosis of pharyngeal gonorrhoea in the last 3 months (n = 9, 30%). Almost all men reported a casual sexual partner in the past three months (n = 28, 93%). Table 2 outlines the demographic characteristics of participants.

**Table 2:** Demographic participant characteristics (N = 30)

Demographics	Total n(%)* or median [IQR]
Age	32 [26–46]
Country of birth	
*Australia*	17 (61)
*Other*	11 (39)
Employment status	
*Employed*	20 (67)
*Not employed*	10 (33)
Past history of gonorrhoea (pharyngeal, urethral or rectal)	
*No*	8 (27)
*Yes*	22 (73)
Pharyngeal gonorrhoea past 3 months	
*No*	20 (69)
*Yes*	9 (30)
*Not tested*	1 (1)
Have a current regular sexual partner	
*No*	5 (17)
*Yes*	25 (83)
Number of current regular partners	1 [1–3]
Number of casual partners last 3 months	5 [2–10]

*percentages may not total 100% due to rounding up

There were no major differences between our sample and the larger GONE study participants on a number of demographic variables (P > 0.05) apart from age. Men who were interviewed for the study were younger than men who were not (P < 0.05). Comparing men who were interviewed by telephone with those interviewed face to face, there were no significant differences in age, employment status, number of casual sexual partners in the last three months and pharyngeal gonorrhoea positivity (P > 0.05), however men who were Australian born were more likely to be interviewed by telephone than men born overseas (P = 0.01).

### Men’s views and knowledge of pharyngeal gonorrhoea

#### Knowledge

Overall, the majority of men (n = 29) had heard of rectal, urethral or pharyngeal gonorrhoea with rectal and pharyngeal gonorrhoea the least well known. Most men (n = 21) attributed their knowledge of pharyngeal gonorrhoea to having had a positive diagnosis in the past or through medical staff who had discussed pharyngeal gonorrhoea when screening for STIs. A few men (n = 3) had heard about pharyngeal gonorrhoea through partners or friends who had been diagnosed in the past.

Table 3 shows example quotes of men’s views and knowledge of pharyngeal gonorrhoea.

**Table 3:** Men’s views and knowledge of pharyngeal gonorrhoea

Men’s views and knowledge of pharyngeal gonorrhoea	Quotes from participants
Knowledge	*Yeah*, *it’s interesting*, *it’s not something that I’d thought about*, *maybe because I’d never had throat based gonorrhoea before*, *but since that diagnosis*, *a lot of people I know have said they’ve had throat based gonorrhoea*. *(Participant 26*, *age 45*, *positive pharyngeal gonorrhoea* )*.
Not a serious STI	*What information I have collected tells me that it’s not serious*. *Certainly my experience of having gonorrhoea in the throat is it’s completely asymptomatic and nobody gave me the indication that it was an urgent matter—it was the case of you get it*, *get it treated and then*, *you know*, *get rid of it…I didn’t have any alarm bells rung by anything or any information I collected (Participant 18*, *age 35*, *positive pharyngeal gonorrhoea)*.
Serious but not as serious as HIV	*I don’t think of it as common but I do think of it as serious*. *I guess I would rank it lower [with] HIV being the most concerning STI that I would talk to my partners about in that are you negative and do you test regularly?(Participant 22*, *age 34*, *negative pharyngeal gonorrhoea)*.
Transmission by saliva or ejaculate	*It would probably be blow jobs…yeah with kissing or blow jobs or anything where there’s contact of saliva or exchange of saliva (Participant 1*, *age 36*, *negative pharyngeal gonorrhoea)*. *Well*, *pre-ejaculate and saliva*, *through talking to the staff here at Melbourne Sexual Health Clinic*, *just common sense*, *and through talking to my GP (Participant 26*, *age 45*, *positive pharyngeal gonorrhoea)*.
Ongoing transmission	*The problem is one person gets cured*, *the other person is getting cured*, *and the other person hasn’t been tested yet*. *And the problem is everyone sleeps with each other and then it’s just this soup that keeps boiling*. *Yeah gonorrhoea soup (Participant 10*, *age 30*, *positive pharyngeal gonorrhoea)*.*It can or cannot give symptoms*, *so you can carry without really knowing that you are [infected](Participant 1*, *age 36*, *negative pharyngeal gonorrhoea)*.
Saliva not implicated in transmission	*I imagined before I read up on it that saliva would deal with the bugs*. *I had* *in my mind that by applying saliva to it that it would kill the bacteria (Participant 25*, *age 67*, *negative pharyngeal gonorrhoea)*.
Treatable with resistance a concern	*What I’m hearing now is that it’s becoming resistant to antibiotics which makes it a bit more serious*. *It’s still a treatable STI so in the realm of STI’s it’s still in the treatable bucket*, *it’s like if I get it- get treated*, *but if you happen to get one of those strains that is antibiotic resistant then it would become a bit more of an issue (Participant 10*, *age 30*, *positive pharyngeal gonorrhoea)*.
Difficulty of diagnosis due to the lack of symptoms	*…the thing that concerns me is the lack of symptoms so you’re not aware if there’s anything wrong (Participant 26*, *age 45*, *negative pharyngeal gonorrhoea)*.

*gonorrhoea test result in the last three months

#### Seriousness of gonorrhoea

The majority of men (n = 26) did not consider pharyngeal gonorrhoea to be a serious STI, particularly due to the ease of treatment access and effectiveness. The few men who did consider it serious (n = 4) still regarded pharyngeal gonorrhoea to be of low level seriousness and as non-life threatening compared to HIV. Men commonly viewed HIV as the STI of the highest concern and against which other risk comparisons were made.

#### Transmission of gonorrhoea

Men attributed a number of risk factors to the transmission of gonorrhoea in general, including multiple sexual partners, casual partners and unprotected anal sex. Over half of men (n = 16) attributed the transmission of pharyngeal gonorrhoea to oral sex or rimming and a couple (n = 2) suggested kissing as an avenue of transmission. Of those men, fluid exchange from those acts, including ejaculate, pre-ejaculate and saliva were identified as the cause of disease transmission. In addition to fluid exchange, the resumption of unprotected sexual activity prior to treatment taking effect and the asymptomatic nature of pharyngeal gonorrhoea were also implicated in transmission by a few. One man thought saliva could prevent transmission as it would act to kill bacteria.

#### Treatment

When asked about treatment of gonorrhoea the majority (n = 19) of men felt it was easy to treat or would be easy to treat because antibiotics were not difficult to obtain or administer and were effective. A few men expressed concerns around possible antibiotic resistance and the efficacy of antibiotic treatment in the long term.

#### Diagnosis

Among men who had pharyngeal gonorrhoea, most (n = 6/9) reported they only became aware of their diagnosis through regular STI testing or by a partner who had recently tested positive for pharyngeal gonorrhoea and had informed them of the diagnosis. A few (n = 3/9) mentioned the lack of symptoms of pharyngeal gonorrhoea contributed to men not being diagnosed or presenting for treatment.

### Men’s views on sexual practices and their willingness to change them to reduce the risk of pharyngeal gonorrhoea

Given recent research indicating possible transmission between anatomical sites by saliva, men were asked about their willingness to change certain sexual practices if it was shown to reduce their risk of pharyngeal gonorrhoea.

#### Kissing

Almost all men (n = 29) reported they enjoyed kissing and considered it a very important act of sexual intimacy, particularly with regular sexual partners. Even with casual partners, kissing was regarded as a prelude to sex, with many men reporting sex may not occur otherwise.

…if I have sex with someone and they don’t want to kiss I just very much miss the experience (Participant 16, age 66, positive pharyngeal gonorrhoea).

The majority (n = 28) of men reported they would not stop kissing in order to reduce pharyngeal gonorrhoea. A few (n = 4) said they would consider not kissing their casual partners. One man said he would not kiss his sexual partners if he knew they had pharyngeal gonorrhoea, but would otherwise always kiss them.

If I did [know he had pharyngeal gonorrhoea] I would not kiss them. If I didn’t I would kiss them (Participant 17, age 24, positive pharyngeal gonorrhoea).

The instances in which men would definitely not kiss were if they felt the partner had bad breath, poor oral hygiene, if they were not attracted to the man or because they did not like kissing anyway. Table 4 shows example quotes of men’s willingness to change sexual practices to reduce pharyngeal gonorrhoea.

**Table 4:** Men’s willingness and preferences toward changing sexual practices to reduce the risk of pharyngeal gonorrhoea

Sexual practice	Would not stop sexual practice	Would consider stopping sexual practice
Kissing	*The sexual act without kissing*, *oh god I couldn’t think of anything worse (Participant 16*, *age 66*, *positive pharyngeal gonorrhoea)*.	*Not with my regular partner*, *no*, *but with my casual partners I have not kissed in the past and it’s been fine*. *In the future*, *if the risk became too great*, *I absolutely*, *wouldn’t do it*. *(Participant 5*, *age 27*, *negative pharyngeal gonorrhoea)*.
Oral sex	*If you want to play the game you have to bring the ball…either that or take your ball and go home*. *I've been told to go home because I won't step up (Participant 27*, *age 44*, *negative pharyngeal gonorrhoea)*.	*Yeah*, *I’d do anything in order to prevent it*, *so yes*, *I would abstain if that was to mean that I wouldn’t get it (Participant 5*, *age 27*, *negative pharyngeal gonorrhoea)*.
Oral sex and condom use	*I tried a condom for oral once and it was horrible*, *the feeling*, *the taste*, *the smell and you don't feel much*, *a waste of time (Participant 7*, *age 46*, *negative pharyngeal gonorrhoea)*.	*Only if I knew he had an infection that he could transmit*. *So if I knew he had gonorrhoea*, *then I would*. *But that’s the only context (Participant 18*, *age*, *31*, *positive pharyngeal gonorrhoea)*.
Rimming	*I probably wouldn’t say no to anyone regular*. *Casuals*, *it would depend on who wanted to do it*. *I don’t think I would ever stop someone who wanted to do it (Participant 10*, *age*, *30*, *age*, *25*, *positive pharyngeal gonorrhoea)*.	*The idea just turns me off*. *I mean I know I’m more than happy to stick my penis in someone’s arse but my tongue is like a different story (Participant 11*, *age*, *21*, *negative pharyngeal gonorrhoea)*.
Saliva use for penile-anal sex	*… it’s a lot less mess to clean up afterwards… the water-based ones are just sticky and tend to irritate a lot of people whereas saliva is quite clean and you don’t have to let’s say*, *interrupt yourself to go and get a bottle*. *Convenient! (Participant 14*, *age 25*, *positive pharyngeal gonorrhoea)*.	*If there’s anal sex*, *or penile-anal penetration*, *I would never use saliva there’d always be lubrication involved (Participant 2*, *age 37*, *negative pharyngeal gonorrhoea)*.
Saliva use for masturbation	*Well considering I wouldn’t stop it for oral sex then I’d probably say no*. *I’ve never had a scenario where someone’s masturbated me and we haven’t engaged in oral sex (Participant 10*, *age 30 positive pharyngeal gonorrhoea)*.	*Oh I don’t like letting them*. *Even if they go to put the fingers in the mouth*, *I’ll stop and reach for the lube*, *I’d rather stop*. *I don’t like it*. *(Participant 23*, *age 61*, *negative pharyngeal gonorrhoea)*.

#### Oral sex

The majority of men (n = 25) reported they regularly engaged in, and enjoyed oral sex with both casual and regular partners and would be unlikely to stop this practice to reduce the risk of pharyngeal gonorrhoea. This was especially the case because most men regarded oral sex as a low risk sexual activity when compared with anal sex.

…. maybe because it’s not as dangerous, not as dangerous for HIV which unfortunately is the virus that everyone actually is scared of. Because nobody is really scared of other diseases, I think that’s why nobody really cares about oral sex, but we should (Participant 3, age 26, negative pharyngeal gonorrhoea).

Oral sex was considered a highly important, pleasurable part of sexual activity and was commonly expected by sexual partners. Of the men, only a few (n = 5) reported they would consider stopping either insertive (participant’s penis in partner’s mouth) or receptive (partner’s penis in participant’s mouth) oral sex to prevent pharyngeal gonorrhoea but only because they did not enjoy it anyway. One man reported he would stop oral sex at saunas as he was more likely to go to saunas for anal sex anyway.

Maybe in a sauna but you don't go there for a blow job you go there for sex (Participant 6, age 36, negative pharyngeal gonorrhoea).

The majority suggested they would definitely not have oral sex if they felt the partner had penile odour or poor hygiene.

… when you have it in front of you, when giving a blow job, when you are in front of the partner’s penis and something seems off, or odd, or mild discolouration or something odd, um, I would usually find a way around it or question my partner (Participant 1, age 21, negative pharyngeal gonorrhoea).

#### Condom use for oral sex

The majority of men (n = 27) reported they would not personally wear a condom for insertive oral sex or ask their partners to wear one for receptive oral sex, as they believed the taste and smell of condoms were undesirable for both themselves and their partner and because it would impair the sensation and pleasure of oral sex. They also felt that asking their sexual partners to use condoms for oral sex would not be acceptable.

I feel like I should be using them and I feel like I should be asking others to use them but I just don’t see it as realistic like I think it would kill the mood too much (Participant 21, age 25, negative pharyngeal gonorrhoea).

Interestingly most men had tried wearing condoms for oral sex at least once in the past and acknowledged their risk of pharyngeal gonorrhoea would be reduced if condoms were used for oral sex. However in general men felt that oral sex was an integral and enjoyable part of sex, of relatively low risk, that condom use for oral sex was not popular in the MSM community, and therefore they were willing to accept the risk of pharyngeal gonorrhoea transmission.

Never considered condoms for oral. The pleasure is more important than the risk (Participant 11, age 21, negative pharyngeal gonorrhoea).

A couple of men suggested they would ‘consider’ using a condom for oral sex with a casual partner to prevent pharyngeal gonorrhoea and one man reported he would ask a partner to wear a condom for receptive oral sex if he knew he had pharyngeal gonorrhoea.

#### Rimming

The majority of men (n = 27) had engaged in insertive (participant’s tongue in partner’s anus) and receptive (partner’s tongue in participant’s anus) rimming at some stage in their sexual lives. Most (n = 24) reported they did not enjoy rimming, in particular insertive rimming often due to a perceived lack of hygiene.

…your mouth and that area [anus] aren't really supposed to meet up with each other…and they always want to kiss afterwards and that just doesn't work (Participant 27, age 44, negative pharyngeal gonorrhoea).

A couple of men reported receptive rimming was not a sexual act they enjoyed.

I can’t go that way, can’t handle it, can’t even think about it. Yeah they [try] to and I usually say no don’t go that way (Participant 16, age 66, positive pharyngeal gonorrhoea).

In general rimming was regarded as a very intimate act that was usually reserved for close or regular partners rather than casual partners.

[It’s] probably something you’d do with a more frequent partner or a boyfriend than I would generally do with randoms…it’s an intimacy thing (Participant 28, age 31, negative pharyngeal gonorrhoea).

Given the general dislike of insertive rimming, most men indicated a willingness to restrict it to very intimate or regular partners or to not practice rimming at all in order to reduce pharyngeal gonorrhoea.

### Saliva as a lubricant for penile-anal sex

The majority of men (n = 28) reported they normally used commercial lubricant for anal sex and did not like to use saliva as a lubricant as it was not lubricating enough and made sex uncomfortable.

…if I’m having receptive anal sex with someone I don’t find using saliva to be particularly comfortable… I would much prefer …to use lube (Participant 26, age 45, positive pharyngeal gonorrhoea).

One man preferred to continue to use saliva as it was easier to clean up after sex. The majority of men were prepared to reduce their use of saliva for anal sex as they did not use it regularly anyway and commercial lubricant was preferable. However as one man noted, saliva was always present, even if a commercial lubricant was used for anal sex, due to other saliva transmitting practices during sexual engagement.

Even without saliva being used for that [anal sex] you’re still putting like fingers in various places and so stuff’s gonna travel around from there and so whether it’s saliva or the lube doing it there’s definitely stuff up for grabs! (Participant 27, age 44, negative pharyngeal gonorrhoea).

### Saliva as a lubricant for masturbation

For the majority of men neither commercial lubricant nor saliva was commonly used for masturbation. As a result most men indicated they would be willing to stop using saliva for masturbation to reduce their risk of pharyngeal gonorrhoea. Saliva was most likely to be used for masturbation only when required to reduce friction if they or their partner was circumcised.

Because if they’re circumcised …they don’t have that extra skin to sort of glide, they need lube when they jack off (Participant 11, age 21, negative pharyngeal gonorrhoea).

Saliva was also likely to be used for scenarios of quick sex when no commercial lubricant was available.

When it has been a casual hook-up it’s fast and …if you’re at a gay beat or something like that, like there’s no lube around or anything so like they’re using their spit to jack them off (Participant 11, age 21, negative pharyngeal gonorrhoea).

One man stated he would sometimes use a lubricant for masturbation however it was a lubricant that he made himself as he did not like or trust the ingredients in commercial lubricant and avoided saliva.

I make my own. One teaspoon of xanthan gum, one cup of boiling water. Um, ‘cause it’s, it doesn’t have all the other extra crap that are in the store bought ones tend to be anaesthetic or something like that. There’s a few people who mix their own but it’s not very common (Participant 27,age 44, negative pharyngeal gonorrhoea).

### Differences between groups of men—willingness to change sexual practices

As part of the study we explored possible differences in the views of MSM by demographic characteristics and mode of interview. There were no major differences between MSM by mode of interview, however there were some differences by age and past history of pharyngeal gonorrhoea.

#### Age

Overall younger men (under the median age of 32) were more likely to report a willingness to change certain sexual practices if it were to reduce their risk of pharyngeal gonorrhoea compared to older men—however they were few in number. More young men reported they would be willing to stop kissing, rimming their partners and consider using condoms for oral sex. Two young men said they would use condoms for oral sex if they knew their partners had pharyngeal gonorrhoea or if they had more evidence to suggest it would prevent pharyngeal gonorrhoea. None of the older men said they would be willing to abstain from oral sex, rimming or kissing or use condoms for oral sex in order to prevent or reduce the risk of pharyngeal gonorrhoea.

#### Past history of pharyngeal gonorrhoea

Men who had a recent diagnosis of pharyngeal gonorrhoea (in the last three months) more commonly reported they would be likely to stop rimming compared to men who had tested negative for pharyngeal gonorrhoea, in particular with casual partners. There were no other thematic differences observed between men who tested positive and men who tested negative for pharyngeal gonorrhoea in terms of their willingness to stop other sexual practices, including oral sex, kissing and using condoms for oral sex, to reduce their risk of pharyngeal gonorrhoea.

### Acceptability of alcohol-containing mouthwash use to reduce the risk of pharyngeal gonorrhoea

MSM were eager to talk at length about their views of mouthwash use in the potential reduction of pharyngeal gonorrhoea.

Just over half of men reported they currently used a mouthwash regularly (between 2 and 6 times per week) and six men reported they used it daily. Of these men, around half used alcohol-containing mouthwash and almost half used alcohol-free mouthwash, with a couple of men unsure of which type they used. Most men used mouthwash for personal hygiene purposes in order to freshen the breath and reduce oral bacteria and did not dilute their mouthwash prior to use.

When asked whether they would be willing to use an alcohol-containing mouthwash as part of their daily oral routine to potentially reduce the risk of pharyngeal gonorrhoea, the majority (n = 28) of men reported they would be willing to do so.

I’d be delighted with something that could I could use perhaps before [oral sex] but probably after to mitigate any issues (Participant 27, age 44, negative pharyngeal gonorrhoea).

A few men reported they were already using it before and/or after having oral sex, either as a form of oral hygiene in preparation for a sexual partner or following sex, to reduce their risk of STI transmission. A couple of men, currently using alcohol-free mouthwash, reported they would consider swapping to alcohol-containing mouthwash if it was shown to be beneficial.

The main reasons men reported they would be willing to use alcohol-containing mouthwash to potentially reduce the risk of pharyngeal gonorrhoea were ease of use and convenience, that they were already using it sometimes as a potential preventative or they would be very willing to try anything new to assist in reducing their risk of pharyngeal gonorrhoea. Table 5 outlines the reasons men would consider using alcohol-containing mouthwash to reduce the risk of pharyngeal gonorrhoea.

**Table 5:** Reasons for using an alcohol-containing mouthwash to reduce pharyngeal gonorrhoea transmission.

Reasons for using an alcohol-containing mouthwash	Quotes from participants
Ease and convenience	.* *.* *.*it wouldn’t be hard to sort of keep a bottle on you*. *I mean sex on premises is a bit more difficult but if I was going to someone’s house or they were coming over*, *it would be quite easy to bring a bottle of Listerine with you (Participant 10*, *age 30*, *positive pharyngeal gonorrhoea)*.
Already uses around sex	*I just keep it in the car*, *like usually 100mL*, *[and] just prior [to oral sex] just one rinse and spit out*. *Afterwards [after oral sex]it will be up to three washes*, *an initial rinse and spit out then another mouthful and gargle and spit out*, *then another rinse to flush out then spit out (Participant 23*, *age 61*, *negative pharyngeal gonorrhoea)*.
Willing to change to alcohol-containing mouthwash	*I prefer to use non-alcoholic mouthwashes as I think that I have a very sensitive mouth and I actually would rather use mouthwash more times than not but that being said if I could see that there was a small benefit to using mouthwash with alcohol then I’d be willing to switch to that to prevent these things (Participant 12*, *age 28*, *positive pharyngeal gonorrhoea)*.

#### Proof of effectiveness

Some men who said they would consider using alcohol-containing mouthwash to reduce pharyngeal gonorrhoea transmission, felt they would like more evidence of its effectiveness before they would definitely use it.

If it was proven to make an impact then yes, absolutely! (Participant 1, age 36, negative pharyngeal gonorrhoea).

#### Concerns about the use of alcohol-containing mouthwash

While alcohol-containing mouthwash was highly acceptable to the majority of men, a few expressed concerns about its potential use in reducing the risk of pharyngeal gonorrhoea including: whether the alcohol would exacerbate inflammation to cuts in the mouth or the gums and therefore increase risk of infection if used shortly before sex, whether there would be a possible risk of oral cancer, and concern that the alcohol might kill the naturally occurring bacteria in the mouth. These men either currently did not use mouthwash at all or used alcohol-free mouthwash. Table 6 shows example quotes of men’s concerns about using alcohol-containing mouthwash to reduce pharyngeal gonorrhoea.

**Table 6:** Men’s concerns about using an alcohol-containing mouthwash to reduce pharyngeal gonorrhoea transmission.

Men’s concerns of using an alcohol-containing mouthwash	Quotes from participants
Oral inflammation and increased risk of infection	*My only concern with mouthwash is similar to my concern with brushing teeth which is … that you shouldn’t brush your teeth or use mouthwash within a certain window period of time before having oral sex because it may cause any cuts … to become inflamed because of the alcohol content and it could mean that there is an increased risk of infection…*.*(Participant 2*, *age 37 negative pharyngeal gonorrhoea)*.
Possible cancer causing effect from alcohol content	*I used to use Listerine but ever since I heard what it does to your mouth I’ve stopped*. *I’ve heard it can actually cause* [oral] *cancer and it’s actually not good for your teeth*, *it sort of erodes away the enamel (Participant 10*, *age 30*, *positive pharyngeal gonorrhoea)*.
Natural bacteria would be destroyed by alcohol	*[Alcohol would] kill the natural oral bacteria which would exacerbate STIs* (*Participant 21*, *age 25*, *negative pharyngeal gonorrhoea)*.

## Discussion

This is the first study to explore MSM’s knowledge of pharyngeal gonorrhoea, their willingness to change certain sexual practices potentially related to pharyngeal gonorrhoea transmission, and acceptability of mouthwash as a possible inhibitor of pharyngeal gonorrhoea. We found pharyngeal gonorrhoea was the least known form of gonococcal infection to participants. Gonorrhoea was generally considered not serious, easy to treat, and with transmission considered by some to be through oral sex and saliva as well as higher numbers of sexual partners. We also found that MSM highly value and enjoy kissing, condom-less oral sex and to a lesser extent receptive rimming and would be highly unlikely to stop these saliva transmitting practices regardless of risk. The only practices men were willing to stop were those they did not enjoy or practice anyway, including receptive rimming. If found to be effective, the use of mouthwash against pharyngeal gonorrhoea was found to be highly acceptable amongst MSM in this study. Our findings suggest that despite most men having some knowledge of gonorrhoea and some attributing transmission to oral sex and saliva, it is very unlikely they will make substantial sexual behaviour changes to reduce their risk of pharyngeal gonorrhoea, however, they would be very likely to use behavioural interventions such as mouthwash if it was found to be effective in reducing their risk.

It is not surprising MSM reported an unwillingness to stop the sexual acts they found pleasurable and an important part of sexual practice in order to reduce pharyngeal gonorrhoea. In Rosenberger’s *et al*, 2011 US study of 24,787 MSM completing an online survey on the characteristics of their most recent sexual encounter, men reported kissing and oral sex were frequent, pleasurable and integral sexual acts [25]. Similarly Prestage *et al*, 2009 in an online survey of 2306 mostly homosexual men in Australia examining men’s understanding of pleasure and how it affects the decisions men make about sex, found kissing and oral sex were considered pleasurable and exciting activities among MSM with rimming reported as less pleasurable [26]. The enjoyment of oral sex among MSM was also reported by Richters *et al* (2003) in a study of 75 Australian MSM, which found oral sex was almost always practiced among MSM and mostly without condoms [41]. In that study most men reported they were not concerned about the risks associated with oral sex, except when there was visible blood or with the presence of ejaculate in the mouth, with HIV acquisition the primary concern when this occurred. Arrington-Sanders et al (2016)[29] report using condoms for oral sex is unfavourable to MSM due to the taste and sensation of the condom, and is therefore unlikely to be considered.

Our findings are consistent with the findings of these studies with most men viewing oral sex as a low risk activity that did not require using a condom, despite attributing saliva and seminal fluids during oral sex, kissing and rimming to the transmission of pharyngeal gonorrhoea.

Men’s enjoyment of these practices overrode any concern about the risk of transmission of pharyngeal gonorrhoea and unless there are issues relating to hygiene, odour or a dislike of a particular sexual practice, men are unlikely to change their sexual practices to reduce the risk of acquiring an STI they largely regard as non-serious and easily treatable. Consistent with Prestage *et al*’s (2009) finding [26] that rimming is less preferred than oral sex, men in our study generally did not enjoy insertive rimming and expressed a willingness to stop this in order to reduce the risk of gonorrhoea. Given insertive rimming has been shown to have an independent association with pharyngeal gonorrhoea [5] targeting insertive rimming as a preventative strategy is likely to be supported by many MSM. However as insertive rimming was not commonly practiced or enjoyed by MSM it is unlikely that this strategy alone would reduce the risk of pharyngeal gonorrhoea transmission.

Despite younger men in our study indicating a greater willingness to change sexual practices to reduce their risk of pharyngeal gonorrhoea, previous studies have reported that young men are also more likely to be diagnosed with pharyngeal gonorrhoea [5, 6]. While younger men may hypothetically report a greater willingness to change sexual practices, it should be noted this may not reflect actual practice or may reflect a degree of social desirability bias when interviewed.

### Strengths and Limitations

The strength of this study is that it is the first study, to our knowledge, to examine MSM’s views and knowledge of pharyngeal gonorrhoea, their willingness to change their current sexual practices and the acceptability of using alcohol-containing mouthwash to reduce the risk of pharyngeal gonorrhoea. Importantly this study has shown MSM are unlikely to change sexual practices they highly enjoy regardless of whether there is evidence to suggest pharyngeal gonorrhoea may be transmitted through these practices. This is an important finding for the development of any future intervention aimed at reducing the risk of pharyngeal gonorrhoea among MSM as most men will prioritise sexual enjoyment over risk, particularly if a sexual act is considered to be of low risk.

Possible STI campaigns could focus on educating MSM on the risk categories of sexual behaviour. For example oral sex was considered low risk among MSM however the literature implicates oral sex and saliva exchange as a source of transmission. Sexual health education might focus on educating men on the risks of oral sex in transmitting gonorrhoea. If mouthwash were found to be effective possible uptake of this intervention may also play a role in reducing transmission, however these results are not yet conclusive.

There are a number of limitations to our study, the main limitation being that our sample included a small number of MSM who were purposively recruited from one sexual health service frequented by higher risk MSM and the majority of which had a past history of a gonorrhoea diagnosis, which may have influenced our results. It is possible therefore that our self-selected sample may be biased and our findings may not be generalizable to the broader community of MSM.

In saying this, our sample included MSM from a broad demographic range, and included men who had tested both positive and negative for pharyngeal gonorrhoea and did not majorly differ from the larger GONE study sample of MSM. A further limitation of the study is that the questions were of a hypothetical nature–it is possible men’s views may have differed in practice, if it became accepted knowledge that pharyngeal gonorrhoea transmission could be reduced through abstaining from sexual acts involving saliva exchange or the use of an alcohol-containing mouthwash. Finally, despite our efforts to minimise the effects of social desirability, it should be noted that it is possible men’s responses around their sexual practices may not be entirely accurate due to social desirability bias.

Understanding how pharyngeal gonorrhoea is acquired in MSM is key to reducing the risk of transmission. Previous research shows gonorrhoea can be detected in saliva by laboratory culture [7, 42], suggesting that gonorrhoea may be transmitted through saliva via sexual activities such as rimming, kissing, and saliva use as a lubricant for anal sex. Results from those studies suggest the pharynx may play a central role in gonorrhoea transmission among MSM. If *N*. *gonorrhoeae* is in saliva then transmission would be possible from mouth to mouth through kissing, from mouth to penis through saliva use for masturbation, and from mouth to anus via rimming. It is unlikely MSM will be willing to stop these practices and therefore it is imperative alternative and acceptable approaches that are likely to be utilised by MSM, are examined. Alcohol-containing mouthwash targeting the throat and reducing the possibility of spread from the pharynx to other sites is an easy and cheap alternative that may prove to be effective and has previously been found to be acceptable among a small sample of MSM [43]. With increasing notifications and concerns of future antibiotic resistance and reluctance for sexual behaviour change among MSM exploring novel strategies to reduce pharyngeal gonorrhoea is of urgent importance.

### Future implications

Gaining an understanding about men’s views and knowledge of pharyngeal gonorrhoea and their willingness to change sexual practices is important in the development of future strategies and interventions aimed at reducing the transmission of pharyngeal gonorrhoea among MSM. The role of saliva in the transmission of pharyngeal gonorrhoea requires further large scale, robust investigation, particularly considering the reluctance of MSM to stop certain sexual activities that may have a high risk of pharyngeal gonorrhoea transmission if saliva is shown to be a factor. The high level of acceptability of mouthwash among men in this study suggests that it is a measure likely to be utilised by MSM if it is shown to be effective in reducing pharyngeal gonorrhoea transmission. However, further clinical and laboratory studies on the efficacy of mouthwash are required.

It is important to consider in clinical practice as well as public health campaigns that MSM may be unlikely to reduce sexual behaviours putting them at a higher risk for pharyngeal gonorrhoea, or to utilise condoms, if the sexual practice is highly enjoyable and an expected part of sexual contact between partners. For these reasons, alternative, innovative strategies MSM are likely to uptake need to be investigated such as the use of alcohol containing mouthwash, which MSM report would be quick, easy and likely to be used if proven to be effective. We are currently conducting a study to further explore the effect of two different mouthwashes on the reduction of pharyngeal gonorrhoea (ACTRN12616000247471).
